# Aggressive behaviour among children and adolescents at a public outpatient psychiatric facility

**DOI:** 10.4102/sajpsychiatry.v31i0.2465

**Published:** 2025-07-08

**Authors:** Helene E. Kritzinger, Buhle Kunene, Mpho Letsatsi, Mthobisi Malewa, Teledi Maputha, Busani Mgabhi, Lisemelo Mphutlane, Johan le Roux, Gina Joubert

**Affiliations:** 1 Department of Psychiatry, Faculty of Health Sciences, University of the Free State, Bloemfontein, South Africa; 2 Department of Biostatistics, Faculty of Health Sciences, University of the Free State, Bloemfontein, South Africa

## Introduction

Aggressive behaviour is recognised as a transdiagnostic symptom and is one of the most common reasons for referral of children and adolescents to psychiatric care facilities.^1^ In South Africa, research has found that the life-time prevalence of aggressive behaviour, such as fighting and bullying, increases with age, rising from 65% during primary school to as high as 89% during adolescence.^2^

Prior exposure to aggression or violence has been identified as a significant risk factor for developing aggressive behaviour during adolescence.^3^ This association could be particularly concerning in South Africa, where high levels of violence are prevalent. A longitudinal study following South African individuals from birth to adulthood, for example, found that only 1% of the sample had not been exposed to violence in various environments such as the home, school, peer groups and the community.^2^

Given the long-term impact of aggression in childhood and adolescence and the societal burden it imposes, early detection and intervention are essential.^1^ Studying aggression within the South African context can provide valuable insights, as aggressive behaviour among children and adolescents in developing countries is understudied compared to developed nations.^4^

This study aimed to determine the exposure to and display of aggression among children and adolescents treated as outpatients at the Free State Psychiatric Complex (FSPC) in Bloemfontein, South Africa.

## Methods

The target population of this quantitative cross-sectional study was children and adolescents aged 4–18 years receiving outpatient treatment at the Child and Adolescent Mental Health Centre (CAMHC) of the FSPC at the time of the study in 2019. The CAMHC is a psychiatric outpatient department that provides multiprofessional services to children and adolescents in the Free State province. Data were collected from patient files and noted on a data form compiled according to information obtained from the literature review performed prior to the study. The data form comprised demographical information, clinical diagnosis, whether the patient displayed aggression, and if so, the type of aggression (e.g. physical aggression, such as hitting or kicking, or verbal aggression, such as shouting or swearing) and where directed. Details regarding previous exposure to aggression were also recorded. A pilot study of the first 15 patient files qualifying for the study indicated that a few answer options needed to be added to the data form. The pilot study cases were included in the main study.

The data were analysed by the Department of Biostatistics, University of the Free State (UFS) using SAS Version 9.4 (SAS Institute Inc.; Cary, NC, United States). Results were summarised by frequencies and percentages (categorical variables) or medians (numerical variables). Subgroups were compared using chi-squared or Fisher’s exact tests (categorical variables) and Kruskal–Wallis tests (numerical variables). The association between exposure to and display of aggression was summarised by relative risks with 95% confidence intervals (CI). The significance level was set at 0.05.

### Ethical considerations

Ethical clearance to conduct this study was obtained from the University of the Free State Faculty of Health Sciences Research Ethics Committee (HSREC) (No. UFS-HSD2019/0543/2910). Permission was also obtained from the Free State Province Department of Health and the Head of the Department of Psychiatry. Patient confidentiality was maintained by ensuring the names of the patients was not noted during data collection. Obtaining informed consent from parents or guardians and assent from the children was not required due to the retrospective nature of the study, using archived patient files as the primary source of information and the researchers not having direct contact with the patients.

## Results

Information of 499 children and adolescents was collected. The majority were male (*n* = 324; 64.9%). The median age of the boys was 11 years (interquartile range [IQR] 8.5–14 years) and of the girls 12 years (IQR 9–14 years). Most of the children and adolescents (*n* = 375; 75.2%) were diagnosed with a psychiatric condition (244 [75.3%] of the boys and 131 [74.9%] of the girls). The most common diagnosis was attention-deficit/hyperactivity disorder (ADHD), which occurred in 49.6% of the boys and 40.6% of the girls.

Nearly two-thirds of the children and adolescents in the study (*n* = 318; 63.7%) displayed aggression, and more than a third (*n* = 182; 36.5%) had previous exposure to aggression. The percentage of girls displaying aggression was close to significantly lower than the boys (58.3% versus 66.7%, *p* = 0.06). However, a significantly higher percentage of the girls had previous exposure to aggression than the boys (49.1% versus 36.5%, *p* < 0.01). In both display of aggressive behaviour and exposure to aggression, the difference observed between boys and girls was regarding physical aggression. No significant differences were observed regarding the median age of children and adolescents displaying different kinds of aggression or exposed to different types of aggression.

In both boys and girls, physical aggression was directed mainly only at other people (64.8% of boys and 56.7% of girls displaying physical aggression). The majority of verbal aggression was also directed only at other people (91.7% of boys and 85.7% of girls displaying verbal aggression). Physical aggression was mainly experienced at home only (67.1% of boys and 76.1% of girls were exposed to physical aggression). Verbal aggression was also mainly experienced at home only (63.3% of boys and 62.5% of girls were exposed to verbal aggression). If exposed to aggression at home, it was mainly from parents only (85.1% of boys and 76.7% of girls exposed to aggression at home). The association between exposure to aggression and displaying aggressive behaviour was only statistically significant in the boys (Table 2, *p* < 0.01).

**TABLE 1:** Types of aggression children and adolescents displayed and types of aggression exposed to.

Type of aggression	Total (*n* = 499)			Boys (*n* = 324)			Girls (*n* = 175)		
	*n*	%	Age*	*n*	%	Age*	*n*	%	Age*
**Displayed**									
None	181	36.3	11.0	108	33.3	12.0	73	41.7	12.0
Physical	179	35.9	11.0	127	39.2	11.0	52	29.7	12.0
Verbal	29	5.8	12.0	18	5.6	11.5	11	6.3	12.0
Both	104	20.8	12.0	66	20.4	11.0	38	21.7	13.0
Type not recorded	6	1.2	-	5	1.5	-	1	0.6	-
**Exposure**									
None	317	63.5	11.0	228	70.4	11.0	89	50.9	11.0
Physical	119	23.8	11.0	63	19.4	11.0	56	32.0	13.0
Verbal	33	6.6	12.0	17	5.2	12.0	16	9.1	12.5
Both	27	5.4	12.0	16	4.9	11.5	11	6.3	12.0
Type not recorded	3	0.6	-	0	0.0	-	3	1.7	-

* , Median age in years.

**TABLE 2:** Association between exposure to aggression and displaying aggressive behaviour.

Exposure to aggression	Aggressive behaviour displayed (Yes)				
	*n*	%	Relative risk	95% CI	*P*
**Total group**					
Yes (*n* = 182)	132	72.5	1.24	1.09; 1.41	< 0.01
No (*n* = 317)	186	58.7	-	-	-
**Boys**					
Yes (*n* = 96)	77	80.2	1.32	1.14; 1.52	< 0.01
No (*n* = 228)	139	61.0	-	-	-
**Girls**					
Yes (*n* = 86)	55	64.0	1.21	0.94; 1.56	0.14
No (*n* = 89)	47	52.8	-	-	-

CI, confidence interval.

## Discussion

Nearly two-thirds of the children and adolescents in the sample displayed aggression. These findings are comparable with previous research highlighting the high prevalence of perpetrating behaviour among children and adolescents in South Africa.^2^ The absence of a significant difference in the display of physical aggression between genders, with comparable rates in both groups, was unexpected given literature suggesting that males are more prone to exhibit physical aggression.^4,5^ However, consistent with existing evidence, verbal aggression showed similar prevalence across genders.^5^

Slightly over a third of the sample group had prior exposure to aggression, with a substantially higher proportion of girls being exposed to physical aggression. These findings were lower than expected, as longitudinal research has shown that more than half of South African children are exposed to violence, such as parental conflict at home, with numbers rising during adolescence.^2^ The lower prevalence reported in this study may be attributed to differences in study design, including potential underreporting and the absence of multiple informants. The gender differences in exposure to aggression aligned with other South African research reporting that females are more likely than males to be exposed to physical and emotional domestic violence,^6^ highlighting a potential vulnerability in this gender group. The prevalence of both physical and verbal aggression within the home highlights the domestic environment as a major source of aggression for South African children and adolescents, similar to previous research.^2,6^

The findings indicated that both boys and girls displayed more aggression if they had been exposed to it previously, although the association was significant only for boys. This highlights the role of contextual factors in the development of aggressive behaviour and aligns with research suggesting that exposure to aggression, particularly in domestic and community settings, contributes to the development of aggressive behaviour.^3^

### Study limitations

Data for this study were collected through a retrospective review of information available in patient files, relying on subjective reports that might have led to underreporting. The study did not include information on different forms of aggression, such as relational aggression, nor did it investigate the function or frequency of the aggressive behaviour, which could have provided additional insight into the phenomenon.

To enhance the robustness of future research in this area, it is recommended to incorporate questionnaires administered to patients, parents and teachers, encompassing observable behaviours in various contexts, including the home and school environments. Such methodological improvements would allow for a more comprehensive understanding of aggressive behaviour within psychiatric populations.

## Conclusion

The study adds to the limited body of research on aggression among children and adolescents who are psychiatric patients in the South African context. This research enhances our understanding of the developmental trajectory and outcomes of aggression as part of a child’s frame of reference. The findings underscore the crucial role of the family unit, suggesting that strengthening family dynamics may effectively mitigate aggressive behaviour among children and adolescents.
